# Atherogenic, cardiometabolic, and adiposity composite indices for identifying prehypertension and hypertension: conventional regression and explainable machine learning

**DOI:** 10.3389/fendo.2026.1924726

**Published:** 2026-08-25

**Authors:** Hao Sun, Yuan Liu

**Affiliations:** 1 Qingdao Municipal Hospital, University of Health and Rehabilitation Sciences, Qingdao, China; 2 Shandong Cancer Hospital and Institute, Shandong First Medical University and Shandong Academy of Medical Sciences, Jinan, China

**Keywords:** atherogenic index of plasma, cardiometabolic index, Chinese visceral adiposity index, hypertension, METS-IR, prehypertension, triglyceride-glucose index

## Abstract

**Background:**

Prehypertension and hypertension are cardiovascular risk states. We examined whether biomarker-derived atherogenic, cardiometabolic, and adiposity indices were associated with blood pressure status in adults undergoing health examinations.

**Methods:**

This hospital-based cross-sectional study included 2, 136 adults from the Health Examination Center of the Second Affiliated Hospital of Shandong First Medical University between 2024 and 2025. Blood pressure was classified as normal, prehypertension, or hypertension. Composite indices were reconstructed from anthropometric, biochemical, and questionnaire variables. Analyses included multivariable regression, quartiles, 3-knot restricted cubic splines, mixture analysis, incremental evaluation, explainable machine learning, subgroup analyses, and sensitivity analyses.

**Results:**

The cohort included 983 participants with normal blood pressure (46.02%), 422 with prehypertension (19.76%), and 731 with hypertension (34.22%). In fully adjusted analyses, CVAI (OR, 2.17 (1.80-2.62); P<0.01), TyG-WC (OR, 1.94 (1.64-2.28); P<0.01), TyG-BMI (OR, 1.93 (1.66-2.26); P<0.01), and METS-IR (OR, 1.91 (1.63-2.24); P<0.01) showed the strongest standardized associations with abnormal blood pressure. The highest quartiles of CVAI, METS-IR, and TyG-WHtR had higher odds than the lowest quartiles (all P for trend <0.01). Cross-validated AUROC improved from 0.80 to 0.83 after adding clinical and index information. SHAP highlighted CVAI, age, fasting glucose, waist circumference, eGFR, and uric acid.

**Conclusions:**

Composite indices integrating visceral adiposity, insulin resistance, and lipid burden were associated with prehypertension and hypertension. They may help characterize cardiometabolic risk clustering but are not substitutes for direct blood pressure measurement or diagnostic evaluation. These findings support association and screening-value inference, not causal or diagnostic claims.

## 1 Introduction

Elevated blood pressure remains one of the most important modifiable determinants of cardiovascular morbidity and mortality worldwide. Large pooled analyses have shown that the global burden of hypertension and inadequate blood pressure control remains substantial across regions and income settings (1, 2). The continuous relation between usual blood pressure and vascular mortality begins below conventional diagnostic thresholds, indicating that risk accumulation is not confined to established hypertension.3 Blood pressure lowering reduces major cardiovascular events, but timely identification of high-risk individuals remains a persistent challenge in routine preventive care (3–5).

Contemporary hypertension guidelines differ in terminology and thresholds, but they consistently recognize the clinical importance of individuals whose blood pressure is above optimal yet below established hypertension (6–8). In hospital-based health-examination settings in China, a pragmatic three-level classification into normal blood pressure, prehypertension or high-normal blood pressure, and hypertension can capture a gradient of cardiometabolic risk while avoiding treatment-based causal interpretations. Such cohorts are especially valuable because anthropometric measurements, fasting biochemical tests, and brief lifestyle questionnaires are obtained systematically and at low incremental cost.

Insulin resistance, dyslipidemia, and visceral adiposity commonly cluster with elevated blood pressure and may contribute through sodium retention, sympathetic activation, endothelial dysfunction, and inflammatory signaling (9, 10). In obesity-associated cardiometabolic states, ectopic adiposity and sleep-disordered breathing may further amplify systemic inflammation and insulin resistance, although these factors were not directly measured in this cohort (11). BMI has long been used to summarize general adiposity, but single routine biomarkers may incompletely characterize these interconnected pathways (12). Composite indices may better summarize biologically coherent risk domains by integrating fasting glucose, triglycerides, HDL cholesterol, body size, waist circumference, and adiposity distribution. These indices are particularly attractive for routine health-examination research because they can be reconstructed from standard measurements without insulin assays, imaging, or experimental procedures.

The triglyceride-glucose index summarizes fasting glucose and triglyceride information and has been proposed as a surrogate marker of insulin resistance (13, 14). Its clinical relevance is supported by comparison with clamp-derived insulin sensitivity (15). METS-IR integrates glycemia, triglycerides, HDL cholesterol, and BMI (16). BRI and WHtR characterize body shape and waist distribution, whereas WWI expresses waist circumference relative to body weight (17–20). LAP and CMI summarize lipid accumulation and cardiometabolic adiposity (21, 22). AIP and the TG/HDL-C ratio reflect an atherogenic lipid phenotype, whereas Castelli risk indices capture cholesterol-fraction balance (23–25). CVAI was developed to characterize visceral adipose dysfunction in Chinese adults (26). Non-HDL cholesterol and remnant cholesterol further represent atherogenic cholesterol burden related to triglyceride-rich lipoprotein metabolism (27–29). Mechanistically, insulin resistance and the metabolic syndrome provide a plausible bridge between these indices and blood pressure elevation (9, 10).

Machine learning can capture nonlinearity and interactions among routine variables, but its added value should be judged against well-specified regression models rather than assumed. Therefore, advanced modeling should be used not only to maximize discrimination but also to examine calibration, clinical utility, and clinical plausibility. SHAP-based interpretation provides a practical approach for summarizing feature contributions in tree-based models, whereas calibration and decision-curve analyses help avoid overinterpreting discrimination alone.

We therefore established an independent hospital-based health-examination cohort and investigated the associations of atherogenic, cardiometabolic, and adiposity composite indices with prehypertension and hypertension. We further compared individual and grouped indices, assessed nonlinear dose-response patterns, quantified incremental screening performance, applied explainable machine learning, and characterized latent metabolic-adiposity phenotypes. All analyses were designed for cross-sectional association and screening-value inference rather than causal or diagnostic validation.

## 2 Materials and methods

### 2.1 Study design and participants

This was a hospital-based cross-sectional study using routine health-examination data combined with a brief questionnaire. The source population comprised 8, 346 health-examination records of adults aged 18 years or older who attended the Health Examination Center of the Second Affiliated Hospital of Shandong First Medical University between June 2024 and December 2025. When repeated examinations were available for the same participant, the record with the most complete clinical and questionnaire information was retained. The following exclusions were applied sequentially: incomplete clinical data (n=4, 086), missing questionnaire data (n=1, 667), self-reported use of glucose-lowering or lipid-lowering medications (n=117), major diseases including malignant tumors (n=274), clinical measurements outside physiologically reasonable ranges or containing impossible values (n=61), and pregnancy (n=5). The final analytic cohort comprised 2, 136 participants (**Supplementary Figure 1**). Written informed consent was obtained from all participants. Observational reporting followed STROBE principles (30). Prediction-model components were interpreted in light of TRIPOD and PROBAST guidance (31–33).

### 2.2 Blood pressure outcomes

The primary outcome was blood pressure status. Blood pressure was measured during the same fasting health-examination visit by trained physicians using an automated upper-arm electronic sphygmomanometer. The specific manufacturer and model were not retained in the analytic database. A uniform standard-sized cuff was used. Participants were measured in the seated position on the dominant arm after at least 5 minutes of rest, with the bladder emptied, back and arm supported, feet flat on the floor, and legs uncrossed. One routine reading was obtained. If the reading was considered markedly discordant with the participant’s self-reported blood pressure history, the participant rested for an additional 5 minutes and blood pressure was measured again; the final reading was retained. No separate documentation of caffeine, smoking, or exercise restriction was available beyond the fasting examination protocol. The procedure incorporated the core rest and positioning principles of the Chinese hypertension guideline, although the routine examination protocol did not universally obtain duplicate measurements and average the readings as recommended in the guideline (34). Participants were classified as having normal blood pressure, prehypertension, or hypertension. Hypertension was defined as systolic blood pressure at least 140 mmHg, diastolic blood pressure at least 90 mmHg, or self-reported known hypertension and/or antihypertensive medication use. Prehypertension was defined as systolic blood pressure 130–139 mmHg and/or diastolic blood pressure 85–89 mmHg without hypertension. Normal blood pressure was defined as values below the prehypertension range. Detailed antihypertensive drug classes, doses, treatment duration, adherence, and separate diuretic use were not available. Secondary outcomes included abnormal blood pressure, defined as prehypertension or hypertension versus normal blood pressure, hypertension versus non-hypertension, pulse pressure, and mean arterial pressure.

### 2.3 Demographic, lifestyle, anthropometric, and laboratory variables

Demographic and questionnaire variables included age, sex, smoking status, alcohol drinking status, and regular exercise. Anthropometric and vital-sign variables included height, weight, BMI, waist circumference, WHtR, WWI, BRI, systolic blood pressure, diastolic blood pressure, heart rate, pulse pressure, and mean arterial pressure. Laboratory variables included fasting plasma glucose, HbA1c, triglycerides, total cholesterol, HDL cholesterol, LDL cholesterol, non-HDL cholesterol, remnant cholesterol, serum creatinine, eGFR, uric acid, and hsCRP.

### 2.4 Construction of derived indices

Derived indices were reconstructed from raw anthropometric and laboratory variables. Lipid variables were used in molar units for lipid ratios and were converted to mg/dL where formulas required conventional units. BMI was calculated as weight (kg)/height (m)^2 (12). WHtR was calculated as waist circumference divided by height (18, 19). BRI was calculated as 364.2 - 365.5 x sqrt[1 - (waist circumference/2pi)^2/(0.5 x height)^2], with waist circumference and height entered in the same length unit (17). WWI was calculated as waist circumference (cm)/sqrt[body weight (kg)] (20). LAP was calculated as (waist circumference - 65) x triglycerides in men and (waist circumference - 58) x triglycerides in women (21).

CVAI was calculated using the sex-specific Chinese equations incorporating age, BMI, waist circumference, triglycerides, and HDL cholesterol: for men, CVAI = -267.93 + 0.68 x age + 0.03 x BMI + 4.00 x waist circumference + 22.00 x log10(triglycerides) - 16.32 x HDL cholesterol; for women, CVAI = -187.32 + 1.71 x age + 4.23 x BMI + 1.12 x waist circumference + 39.76 x log10(triglycerides) - 11.66 x HDL cholesterol (26). TyG was calculated as ln[triglycerides (mg/dL) x fasting plasma glucose (mg/dL)/2] (13, 14). TyG-BMI and TyG-WC were calculated by multiplying TyG by BMI and waist circumference, respectively (13–15). TyG-WHtR and TyG-WWI were calculated by multiplying TyG by WHtR and WWI, respectively (18–20). METS-IR was calculated as ln[(2 x fasting plasma glucose [mg/dL]) + triglycerides (mg/dL)] x BMI/ln[HDL cholesterol (mg/dL)] (16).

AIP was calculated as log10(triglycerides/HDL cholesterol), and TG/HDL-C was calculated as triglycerides divided by HDL cholesterol (23, 24). TC/HDL-C and LDL-C/HDL-C were calculated as total cholesterol divided by HDL cholesterol and LDL cholesterol divided by HDL cholesterol, respectively (25). AC was calculated as (total cholesterol - HDL cholesterol)/HDL cholesterol (25). Non-HDL cholesterol was calculated as total cholesterol - HDL cholesterol (27). Remnant cholesterol was calculated as total cholesterol - LDL cholesterol - HDL cholesterol (28, 29). CMI was calculated as WHtR x triglycerides/HDL cholesterol (22). All ratios used internally consistent lipid units within each formula.

### 2.5 Conventional regression, nonlinear, and mixture analyses

The distributions of continuous variables were evaluated using histograms, quantile-quantile plots, skewness, and kurtosis, and homogeneity of variance was assessed using Levene’s test. Approximately symmetric continuous variables were summarized as mean (SD), whereas markedly skewed variables, defined by an absolute skewness greater than 1 together with visual distributional assessment, were summarized as median (interquartile range). Between-group comparisons used one-way analysis of variance when variance homogeneity was acceptable, Welch’s analysis of variance when it was violated, and the Kruskal-Wallis test for markedly skewed variables. Categorical variables were summarized as No. (%) and compared using chi-square tests. AIP, which is intrinsically log10-transformed, was approximately symmetric in this dataset (skewness, 0.04) and was therefore presented as mean (SD). hsCRP was log-transformed in multivariable models. All displayed P values were two-sided. Continuous indices were standardized, and odds ratios were expressed per 1-SD increment. Logistic regression was performed separately for each index to reduce collinearity among composite indicators. Models were sequentially adjusted for demographic variables, lifestyle variables, heart rate, HbA1c, LDL cholesterol, eGFR, uric acid, and log-transformed hsCRP. Restricted cubic splines with 3 knots located at the 10th, 50th, and 90th percentiles were used to evaluate dose-response associations, with overall and nonlinearity P values reported (35). Quantile-based mixture analyses estimated the joint association of average quartile scores within atherogenic, cardiometabolic, adiposity, and integrated index domains. These mixture analyses were interpreted descriptively and not as causal mixture effects.

### 2.6 Incremental discrimination, calibration, clinical utility, and machine learning

Incremental screening performance was evaluated using stratified 5-fold cross-validated logistic models. Model domains included a basic demographic-lifestyle model, a conventional-biomarker model, atherogenic-index model, cardiometabolic-index model, adiposity-index model, integrated novel-index model, and full model. Metrics included AUROC, PR-AUC, accuracy, sensitivity, specificity, precision, F1 score, Brier score, calibration intercept, calibration slope, Hosmer-Lemeshow P value, and decision-curve net benefit. AUROC confidence intervals and comparisons were estimated using paired bootstrap resampling principles and interpreted in light of DeLong-type correlated ROC comparison (36). Calibration and clinical utility were evaluated using calibration plots and decision-curve analysis (37, 38). Quantile-based mixture analysis was interpreted by analogy to published g-computation mixture methods (39).

Representative supervised learning models included baseline logistic regression, full logistic regression, elastic-net logistic regression, random forest, gradient boosting, and XGBoost (40, 41). The dataset was split into training and testing sets using stratification by abnormal blood pressure status. Preprocessing was performed within the training workflow to reduce information leakage. SHAP analysis was used to summarize global feature contributions in a representative tree-based model (42, 43).

### 2.7 Unsupervised phenotyping, subgroup, and sensitivity analyses

Gaussian mixture modeling was rerun using age, BMI, waist circumference, WHtR, AIP, CMI, CVAI, METS-IR, TyG-WHtR, BRI, WWI, eGFR, uric acid, and log-transformed hsCRP to identify latent metabolic-adiposity phenotypes. All inputs were standardized. Full-covariance solutions with 2 to 4 components were fitted using 20 random initializations and a fixed random seed of 42; the solution with the lowest Bayesian information criterion was selected. Phenotype labels were ordered from the most favorable to the most adverse mean standardized profile, with eGFR reverse-oriented in the ordering score; blood pressure outcomes were not used to define or order the phenotypes. Subgroup analyses used TyG-WHtR as a representative integrated metabolic-adiposity index. Sensitivity analyses repeated the primary association models after excluding participants with hsCRP >10 mg/L, after excluding one implausible record with systolic blood pressure lower than diastolic blood pressure, and after excluding participants with self-reported known hypertension and/or antihypertensive medication use. Linear models assessed continuous systolic blood pressure, diastolic blood pressure, and mean arterial pressure as secondary outcomes.

## 3 Results

### 3.1 Baseline characteristics

The final cohort included 2, 136 adults aged 18–86 years, of whom 983 (46.02%) had normal blood pressure, 422 (19.76%) had prehypertension, and 731 (34.22%) had hypertension. The mean (SD) age was 47.66 (14.70) years, and 1, 130 participants (52.90%) were male. Baseline characteristics are shown in **Table 1**. Triglycerides, remnant cholesterol, the TG/HDL-C ratio, hsCRP, CMI, and LAP were markedly right-skewed and are presented as median (interquartile range); AIP was approximately symmetric after its intrinsic logarithmic transformation and is presented as mean (SD). Across normal blood pressure, prehypertension, and hypertension categories, age, BMI, waist circumference, WHtR, WWI, BRI, fasting plasma glucose, HbA1c, triglycerides, LDL cholesterol, non-HDL cholesterol, remnant cholesterol, uric acid, hsCRP, TyG derivatives, AIP, CMI, LAP, CVAI, and METS-IR increased progressively, whereas HDL cholesterol decreased; all corresponding overall P values were <0.01.

**Table 1:** Baseline characteristics according to blood pressure status.

Variable	Overall	Normal	Prehypertension	Hypertension	P value
**Age**, **years**	47.66 (14.70)	40.04 (12.22)	48.90 (11.70)	57.20 (13.48)	<0.01
**Height**, **cm**	164.79 (7.94)	163.73 (7.97)	165.09 (7.99)	166.04 (7.68)	<0.01
**Weight**, **kg**	64.58 (10.70)	60.82 (9.88)	65.55 (10.27)	69.07 (10.15)	<0.01
**Body mass index**, **kg/m²**	23.69 (2.94)	22.62 (2.80)	23.97 (2.73)	24.98 (2.69)	<0.01
**Waist circumference**, **cm**	83.30 (8.66)	79.42 (7.88)	84.26 (7.74)	87.97 (7.66)	<0.01
**Waist-to-height ratio**	0.51 (0.05)	0.49 (0.05)	0.51 (0.05)	0.53 (0.05)	<0.01
**Weight-adjusted waist index**	10.40 (0.65)	10.22 (0.63)	10.45 (0.63)	10.62 (0.61)	<0.01
**Body roundness index**	3.52 (0.97)	3.14 (0.88)	3.61 (0.88)	3.99 (0.94)	<0.01
**Systolic blood pressure**, **mmHg**	124.27 (14.95)	113.86 (9.85)	128.94 (9.18)	135.57 (13.72)	<0.01
**Diastolic blood pressure**, **mmHg**	78.25 (8.84)	73.49 (6.84)	80.69 (6.81)	83.23 (8.94)	<0.01
**Heart rate**, **beats/min**	72.76 (7.53)	72.15 (7.44)	73.25 (7.43)	73.31 (7.66)	<0.01
**Pulse pressure**, **mmHg**	46.02 (14.30)	40.37 (10.53)	48.25 (13.78)	52.34 (15.92)	<0.01
**Mean arterial pressure**, **mmHg**	93.59 (9.01)	86.94 (6.24)	96.78 (4.11)	100.68 (7.72)	<0.01
**Fasting plasma glucose**, **mmol/L**	5.30 (0.80)	4.90 (0.66)	5.33 (0.67)	5.82 (0.75)	<0.01
**HbA1c**, **%**	5.42 (0.40)	5.24 (0.33)	5.42 (0.34)	5.66 (0.40)	<0.01
**Total cholesterol**, **mmol/L**	4.99 (0.85)	4.67 (0.74)	5.05 (0.77)	5.38 (0.86)	<0.01
**Triglycerides**, **mmol/L**	1.55 (1.05-2.25)	1.27 (0.88-1.75)	1.60 (1.15-2.26)	1.99 (1.44-2.80)	<0.01
**HDL cholesterol**, **mmol/L**	1.29 (0.22)	1.35 (0.21)	1.28 (0.22)	1.21 (0.21)	<0.01
**LDL cholesterol**, **mmol/L**	2.76 (0.61)	2.54 (0.57)	2.81 (0.55)	3.03 (0.59)	<0.01
**Non-HDL cholesterol**, **mmol/L**	3.70 (0.92)	3.32 (0.79)	3.77 (0.84)	4.17 (0.91)	<0.01
**Remnant cholesterol**, **mmol/L**	0.85 (0.60-1.16)	0.71 (0.52-0.98)	0.87 (0.65-1.16)	1.03 (0.77-1.40)	<0.01
**TG/HDL-C ratio**	1.21 (0.77-1.87)	0.95 (0.61-1.42)	1.23 (0.83-1.91)	1.62 (1.14-2.52)	<0.01
**TC/HDL-C ratio**	4.03 (1.21)	3.57 (0.90)	4.10 (1.17)	4.61 (1.32)	<0.01
**LDL-C/HDL-C ratio**	2.24 (0.76)	1.94 (0.60)	2.29 (0.72)	2.60 (0.81)	<0.01
**Atherogenic coefficient**	3.03 (1.21)	2.57 (0.90)	3.10 (1.17)	3.61 (1.32)	<0.01
**Serum creatinine**, **µmol/L**	75.42 (11.46)	70.38 (10.19)	76.46 (10.12)	81.58 (10.59)	<0.01
**eGFR**, **mL/min/1.73 m²**	95.81 (13.47)	101.74 (12.00)	95.35 (11.68)	88.09 (12.29)	<0.01
**Uric acid**, **µmol/L**	325.78 (65.37)	308.58 (64.20)	330.36 (63.60)	346.25 (61.58)	<0.01
**hsCRP**, **mg/L**	1.09 (0.64-1.88)	0.91 (0.54-1.57)	1.17 (0.69-2.09)	1.32 (0.80-2.13)	<0.01
**TyG index**	8.77 (0.66)	8.49 (0.62)	8.81 (0.58)	9.12 (0.57)	<0.01
**TyG-BMI**	209.14 (38.62)	193.16 (35.08)	212.28 (34.86)	228.81 (35.62)	<0.01
**TyG-WC**	734.54 (121.92)	677.33 (107.29)	745.60 (106.71)	805.09 (109.35)	<0.01
**TyG-WHtR**	4.46 (0.71)	4.14 (0.64)	4.52 (0.61)	4.85 (0.65)	<0.01
**TyG-WWI**	91.33 (10.23)	86.83 (9.35)	92.13 (8.80)	96.93 (9.21)	<0.01
**Atherogenic index of plasma**	0.08 (0.29)	-0.03 (0.28)	0.10 (0.27)	0.22 (0.27)	<0.01
**Cardiometabolic index**	0.62 (0.37-0.99)	0.46 (0.28-0.71)	0.64 (0.41-1.00)	0.87 (0.58-1.35)	<0.01
**Lipid accumulation product**	32.89 (18.10-56.93)	22.30 (12.37-39.15)	34.61 (21.21-57.71)	49.53 (31.62-79.44)	<0.01
**Chinese visceral adiposity index**	84.55 (44.02)	59.48 (37.83)	90.52 (34.28)	114.81 (35.77)	<0.01
**METS-IR**	35.77 (7.07)	32.91 (6.15)	36.30 (6.56)	39.31 (6.83)	<0.01
**Sex**					<0.01
Female	1006 (47.10)	556 (56.56)	192 (45.50)	258 (35.29)	
Male	1130 (52.90)	427 (43.44)	230 (54.50)	473 (64.71)	
**Smoking status**					<0.01
Current	504 (23.60)	180 (18.31)	118 (27.96)	206 (28.18)	
Former	159 (7.44)	46 (4.68)	25 (5.92)	88 (12.04)	
Never	1473 (68.96)	757 (77.01)	279 (66.11)	437 (59.78)	
**Alcohol drinking status**					<0.01
Current	609 (28.51)	237 (24.11)	134 (31.75)	238 (32.56)	
Former	150 (7.02)	45 (4.58)	31 (7.35)	74 (10.12)	
Never	1377 (64.47)	701 (71.31)	257 (60.90)	419 (57.32)	
**Regular exercise**					0.99
No	1241 (58.10)	572 (58.19)	246 (58.29)	423 (57.87)	
Yes	895 (41.90)	411 (41.81)	176 (41.71)	308 (42.13)	

Approximately symmetric continuous variables are presented as mean (SD), markedly skewed variables as median (interquartile range), and categorical variables as No. (%). P values were calculated using one-way analysis of variance, Welch’s analysis of variance, the Kruskal-Wallis test, or the chi-square test, as appropriate. AIP was approximately symmetric after its intrinsic log10 transformation and is presented as mean (SD). All P values are two-sided. Bold text denotes variable names and categorical-variable headings only and does not indicate statistical significance.

The source population included 8, 346 adult health-examination records. After the sequential exclusions shown in **Supplementary Figures 1**, **2**, 136 participants remained, and no variable had missing values in the final analytic dataset. Undiagnosed hypertension was present in 285 participants (13.34%). One record had systolic blood pressure lower than diastolic blood pressure; this record was retained in the primary blood pressure-category analysis because the category was defined by the database, and a sensitivity analysis excluding it showed materially unchanged results.

### 3.2 Fully adjusted associations with abnormal blood pressure and hypertension

In fully adjusted models, nearly all atherogenic, cardiometabolic, and adiposity indices were positively associated with abnormal blood pressure (**Table 2**). The strongest associations were observed for CVAI (OR per 1-SD, 2.17 (1.80-2.62); P<0.01), TyG-WC (OR, 1.94 (1.64-2.28); P<0.01), TyG-BMI (OR, 1.93 (1.66-2.26); P<0.01), METS-IR (OR, 1.91 (1.63-2.24); P<0.01), waist circumference (OR, 1.81 (1.55-2.11); P<0.01), TyG-WHtR (OR, 1.76 (1.52-2.05); P<0.01), and BMI (OR, 1.85 (1.60-2.14); P<0.01). Atherogenic indices also remained significant, including AIP (OR, 1.49 (1.29-1.71); P<0.01), non-HDL cholesterol (OR, 1.65 (1.29-2.11); P<0.01), and remnant cholesterol (OR, 1.31 (1.15-1.50); P<0.01).

**Table 2:** Fully adjusted associations of representative indices with abnormal blood pressure and hypertension.

Domain	Index	Abnormal BP		Hypertension	
		OR (95% CI)	P value	OR (95% CI)	P value
Atherogenic	Atherogenic index of plasma	1.49 (1.29-1.71)	<0.01	1.55 (1.34-1.80)	<0.01
Cardiometabolic	Cardiometabolic index	1.46 (1.23-1.72)	<0.01	1.36 (1.18-1.56)	<0.01
Cardiometabolic	Chinese visceral adiposity index	2.17 (1.80-2.62)	<0.01	2.08 (1.72-2.52)	<0.01
Cardiometabolic	METS-IR	1.91 (1.63-2.24)	<0.01	1.84 (1.57-2.16)	<0.01
Cardiometabolic	TyG-BMI	1.93 (1.66-2.26)	<0.01	1.95 (1.66-2.28)	<0.01
Cardiometabolic	TyG-WC	1.94 (1.64-2.28)	<0.01	1.94 (1.64-2.30)	<0.01
Cardiometabolic	TyG-WHtR	1.76 (1.52-2.05)	<0.01	1.78 (1.53-2.08)	<0.01
Adiposity	Body roundness index	1.57 (1.37-1.80)	<0.01	1.53 (1.33-1.75)	<0.01
Adiposity	Weight-adjusted waist index	1.15 (1.02-1.29)	0.02	1.14 (1.00-1.28)	0.04
Adiposity	Lipid accumulation product	1.59 (1.35-1.87)	<0.01	1.50 (1.31-1.72)	<0.01
Adiposity	Body mass index, kg/m²	1.85 (1.60-2.14)	<0.01	1.83 (1.57-2.12)	<0.01
Adiposity	Waist circumference, cm	1.81 (1.55-2.11)	<0.01	1.76 (1.50-2.07)	<0.01
Atherogenic	Non-HDL cholesterol, mmol/L	1.65 (1.29-2.11)	<0.01	1.65 (1.31-2.08)	<0.01
Atherogenic	Remnant cholesterol, mmol/L	1.31 (1.15-1.50)	<0.01	1.31 (1.16-1.49)	<0.01

Odds ratios are expressed per 1-SD increment. Models adjusted for age, sex, smoking status, alcohol drinking status, regular exercise, heart rate, HbA1c, LDL cholesterol, eGFR, uric acid, and log-transformed hsCRP.

Associations were similar when hypertension was modeled against non-hypertension. For hypertension, CVAI (OR, 2.08 (1.72-2.52); P<0.01), TyG-BMI (OR, 1.95 (1.66-2.28); P<0.01), TyG-WC (OR, 1.94 (1.64-2.30); P<0.01), METS-IR (OR, 1.84 (1.57-2.16); P<0.01), and TyG-WHtR (OR, 1.78 (1.53-2.08); P<0.01) remained prominent.

### 3.3 Quartile and nonlinear analyses

Quartile analyses supported graded associations between representative indices and abnormal blood pressure (**Table 3**). Participants in the highest CVAI quartile had an abnormal blood pressure prevalence of 87.64% and an adjusted OR of 4.97 (3.17-7.79) relative to the lowest quartile (P<0.01; P for trend <0.01). Similar graded associations were observed for METS-IR (Q4 vs Q1 OR, 4.68 (3.16-6.93); P<0.01), TyG-WHtR (OR, 4.13 (2.84-6.02); P<0.01), BRI (OR, 2.83 (2.00-4.01); P<0.01), and LAP (OR, 3.65 (2.52-5.29); P<0.01).

**Table 3:** Highest-quartile associations of representative indices with abnormal blood pressure.

Index	Q4 events/participants	Q4 event rate, %	OR (95% CI)	P value	P for trend
Atherogenic index of plasma	399/530	75.28	2.56 (1.77-3.69)	<0.01	<0.01
Cardiometabolic index	407/533	76.36	2.70 (1.87-3.91)	<0.01	<0.01
Chinese visceral adiposity index	468/534	87.64	4.97 (3.17-7.79)	<0.01	<0.01
METS-IR	406/534	76.03	4.68 (3.16-6.93)	<0.01	<0.01
TyG-WHtR	419/530	79.06	4.13 (2.84-6.02)	<0.01	<0.01
Body roundness index	404/532	75.94	2.83 (2.00-4.01)	<0.01	<0.01
Weight-adjusted waist index	372/534	69.66	1.33 (0.97-1.83)	0.08	0.06
Lipid accumulation product	412/534	77.15	3.65 (2.52-5.29)	<0.01	<0.01

Each row compares the highest quartile with the lowest quartile. Models were adjusted as in **Table 2**.

**Figure 1:**
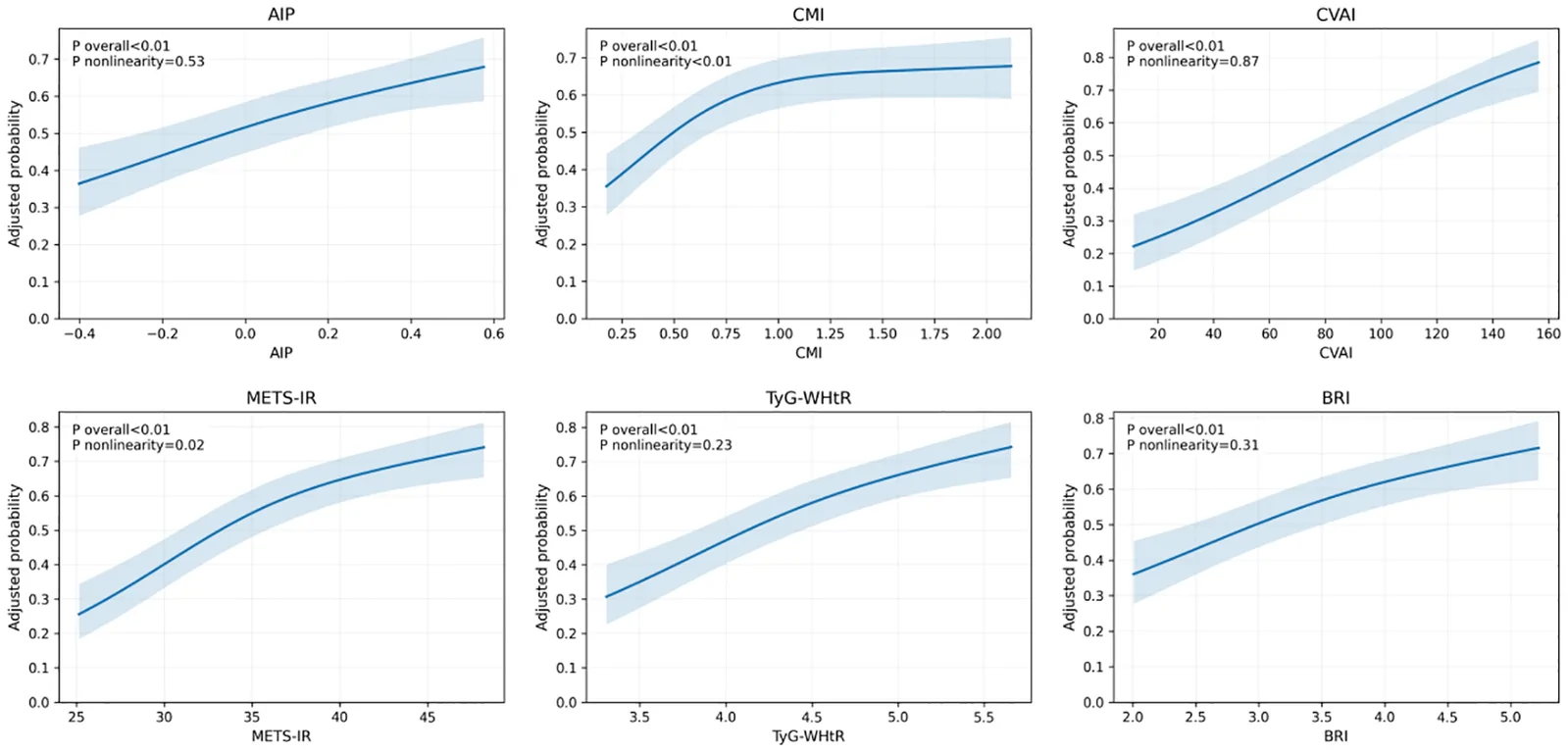
Restricted cubic spline curves with 3 knots for representative indices and abnormal blood pressure. Knots were located at the 10th, 50th, and 90th percentiles. Curves show adjusted probabilities with 95% confidence intervals over the 5th to 95th percentile range.

Restricted cubic splines with 3 knots showed significant overall associations for AIP, CMI, CVAI, METS-IR, TyG-WHtR, and BRI (all overall P values <0.01; **Figure 1**). Evidence for nonlinearity was observed for CMI (P for nonlinearity <0.01), while the remaining indices showed predominantly graded positive patterns.

### 3.4 Mixture analyses and incremental screening performance

Quantile-based mixture analyses supported positive joint associations for atherogenic, cardiometabolic, adiposity, and integrated novel-index domains (**Table 4**). For abnormal blood pressure, the adjusted ORs were 1.43 for the atherogenic mixture, 1.76 for the cardiometabolic mixture, 1.63 for the adiposity mixture, and 1.72 for the integrated novel-index mixture (all P values <0.01). Similar positive associations were observed for hypertension.

**Table 4:** Quantile-based mixture analyses.

Outcome	Mixture	OR (95% CI)	P value
Abnormal BP vs normal	Atherogenic mixture	1.43 (1.21-1.68)	<0.01
	Cardiometabolic mixture	1.76 (1.53-2.03)	<0.01
	Adiposity mixture	1.63 (1.42-1.86)	<0.01
	Integrated novel-index mixture	1.72 (1.49-1.99)	<0.01
Hypertension vs non-hypertension	Atherogenic mixture	1.61 (1.36-1.92)	<0.01
	Cardiometabolic mixture	1.82 (1.57-2.12)	<0.01
	Adiposity mixture	1.60 (1.39-1.85)	<0.01
	Integrated novel-index mixture	1.77 (1.52-2.07)	<0.01

Estimates represent adjusted odds ratios for the average quartile score across components in the specified index domain. Analyses are descriptive and not causal mixture estimates.

**Figure 2:**
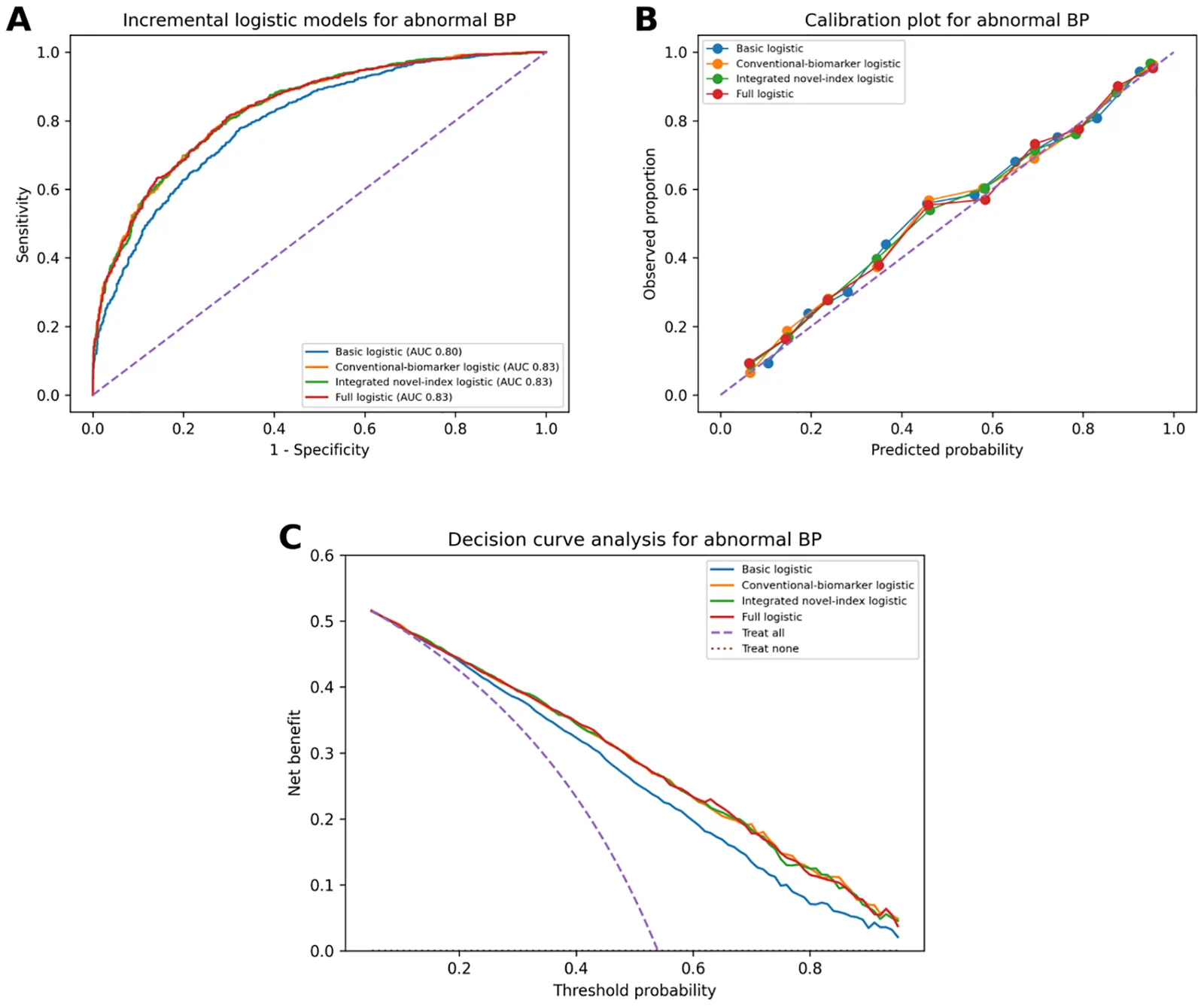
Incremental model performance for identifying abnormal blood pressure. Panel **(A)** shows ROC curves, panel **(B)** shows calibration, and panel **(C)** shows decision-curve analysis.

Cross-validated incremental model performance is shown as a consolidated graphical summary in **Figure 2**. For abnormal blood pressure, the basic demographic-lifestyle model achieved an AUROC of 0.80 (0.78-0.82), and the full logistic model achieved an AUROC of 0.83 (0.82-0.85), corresponding to an absolute AUROC increase of 0.03. Calibration and decision-curve results are presented descriptively without assigning a qualitative performance category because no prespecified threshold for terms such as ‘moderate’ was used.

### 3.5 Supervised machine learning, SHAP interpretation, and unsupervised phenotypes

**Figure 3:**
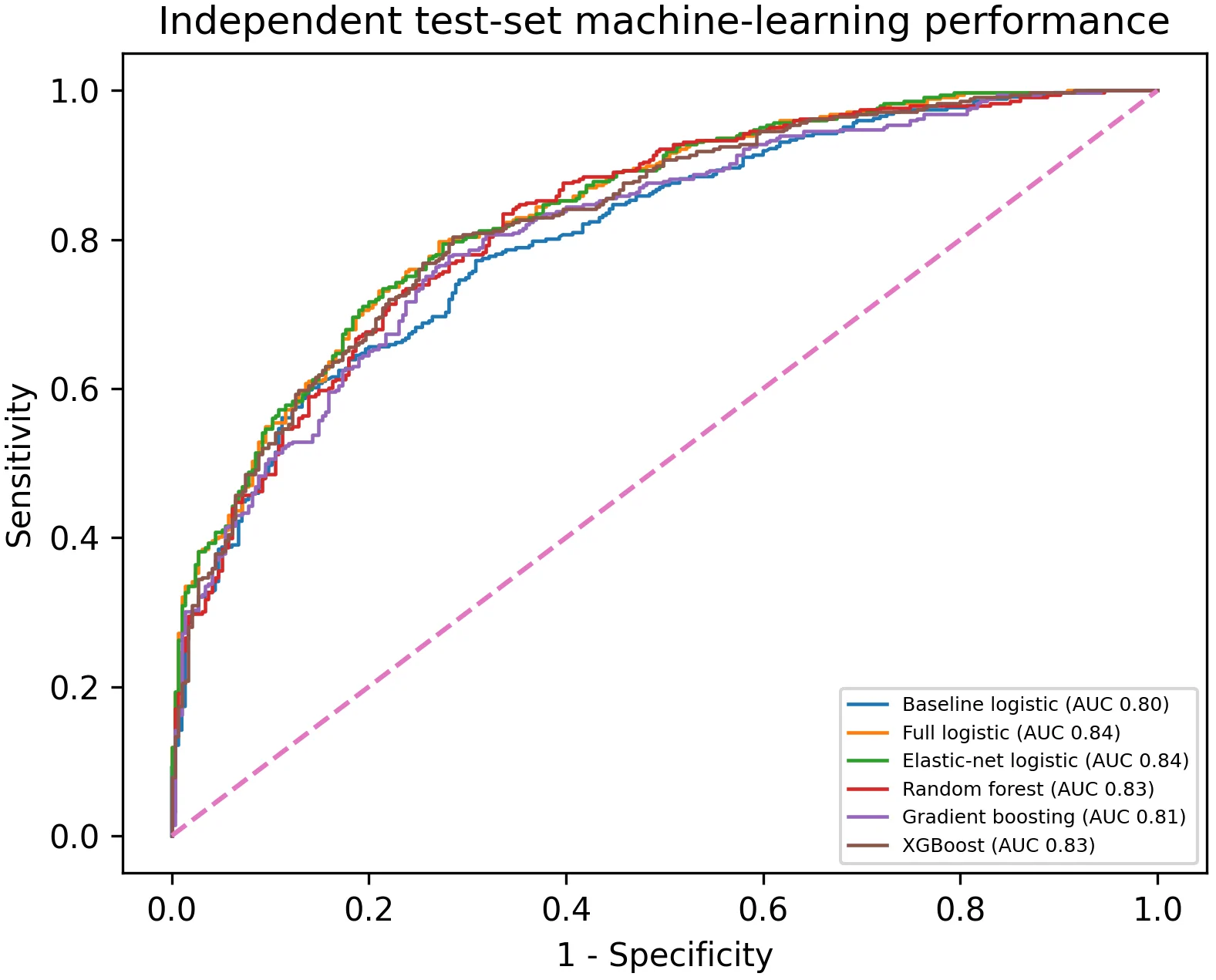
Independent test-set ROC curves for representative regression and machine-learning models.

**Figure 4:**
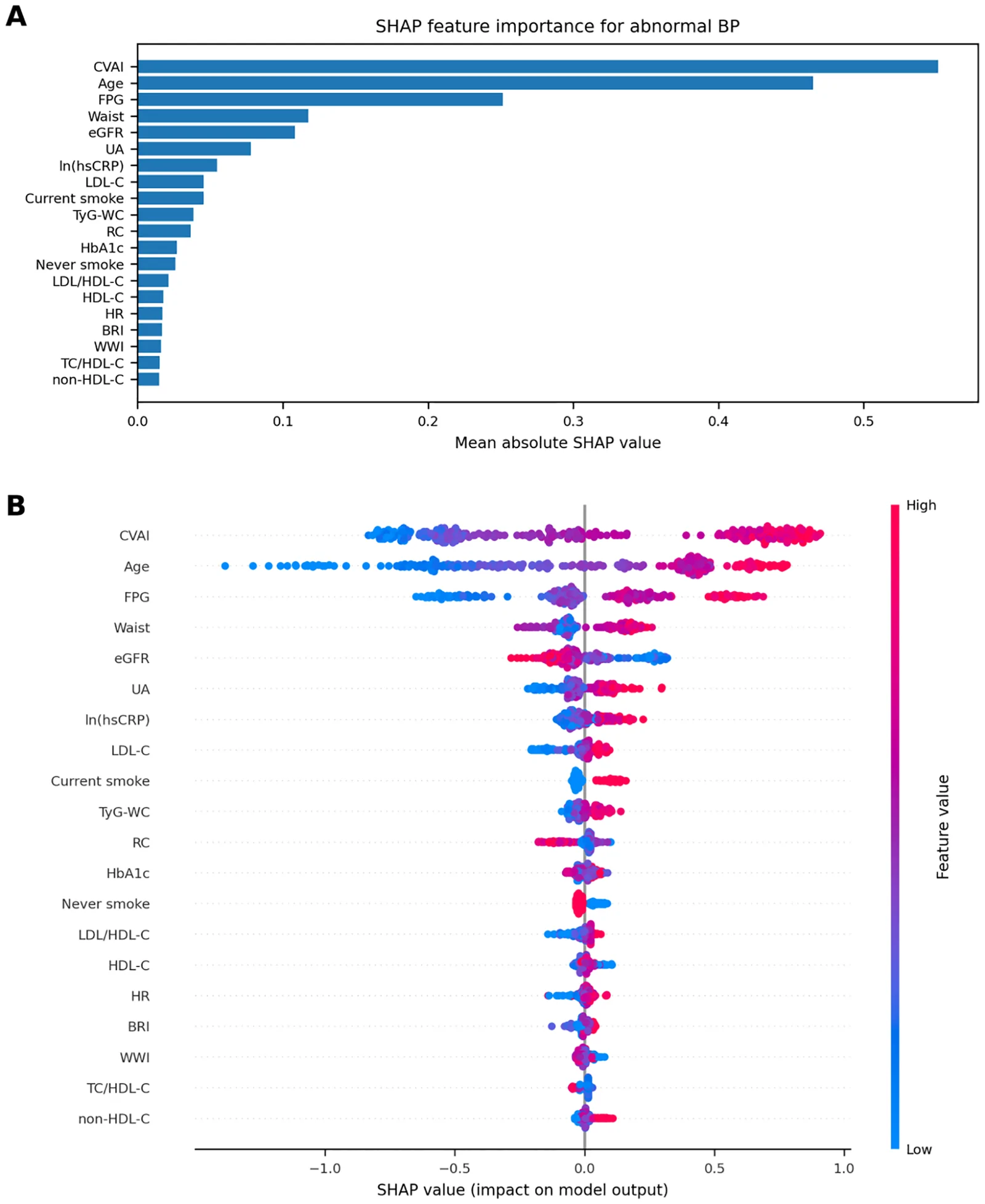
SHAP explainability for the representative tree-based model. Panel **(A)** (upper) shows global feature importance; panel **(B)** (lower) shows the SHAP summary distribution.

Independent test-set machine-learning results are shown in **Figure 3**. SHAP explainability from the representative tree-based model identified cardiometabolic, adiposity, lipid, and hemodynamic variables as the leading contributors to model output (**Figure 4**). Full logistic regression achieved an AUROC of 0.84 (0.81-0.87), elastic-net logistic regression achieved 0.84 (0.81-0.87), and XGBoost achieved 0.83 (0.80-0.86). Although some full-feature models improved over the baseline logistic model, differences among the best-performing models were modest.

**Figure 5:**
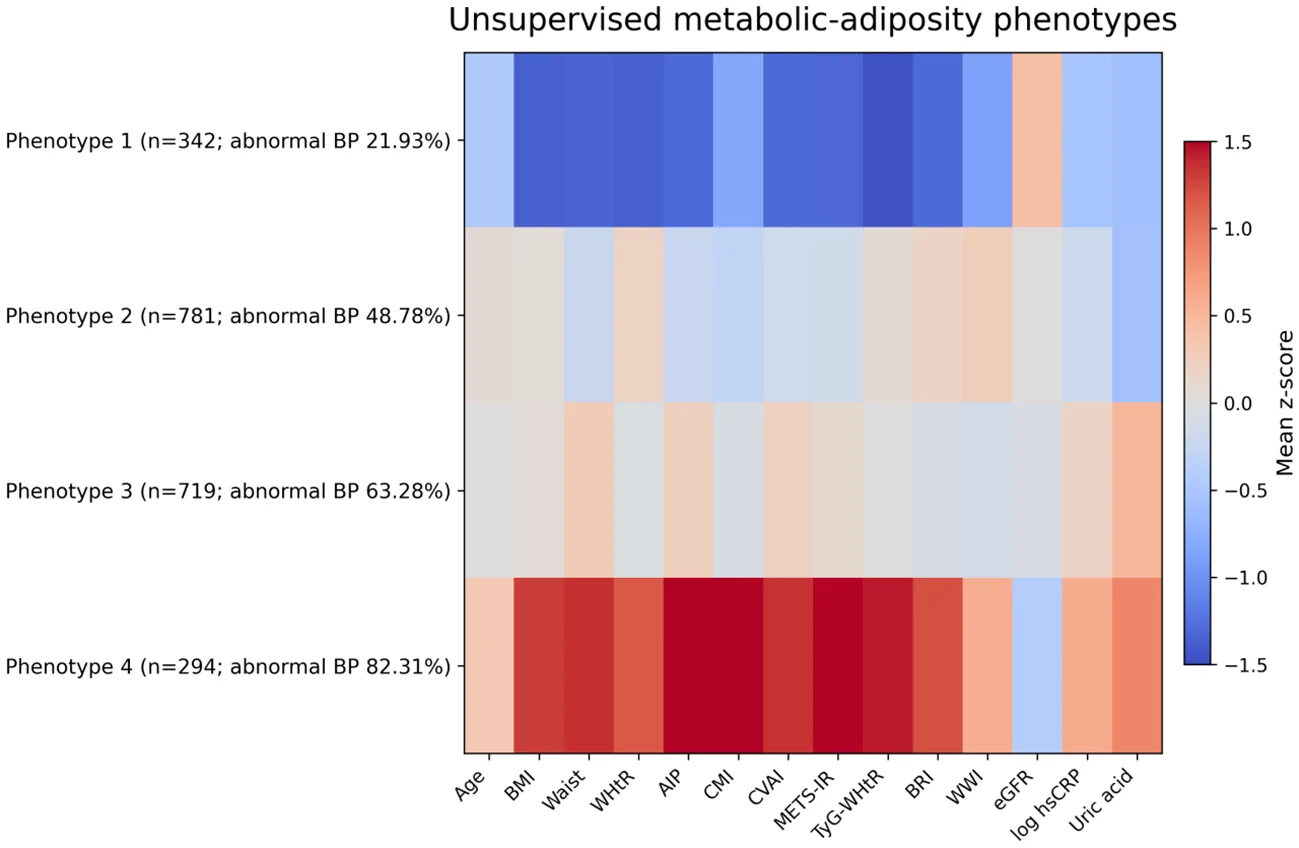
Heatmap of the rerun unsupervised metabolic-adiposity phenotype profiles. Y-axis labels show the number of participants and the prevalence of abnormal blood pressure in each phenotype. Corresponding event counts and adjusted odds ratios are provided in **Table 5**.

In the rerun Gaussian mixture analysis, Bayesian information criterion values were 12, 526.63 for 2 components, 8, 827.64 for 3 components, and -158.60 for 4 components; therefore, the 4-phenotype solution was selected. The phenotypes represented progressively more adverse metabolic-adiposity profiles (**Figure 5**). Abnormal blood pressure was present in 75 of 342 participants (21.93%) in phenotype 1, 381 of 781 (48.78%) in phenotype 2, 455 of 719 (63.28%) in phenotype 3, and 242 of 294 (82.31%) in phenotype 4 (**Table 5**). Compared with phenotype 1, the fully adjusted odds ratios for abnormal blood pressure were 1.88 (1.25-2.84), 3.16 (2.03-4.92), and 4.83 (2.83-8.23) for phenotypes 2, 3, and 4, respectively (all P<0.01).

**Table 5:** Phenotype-specific prevalence and adjusted associations with abnormal blood pressure.

Phenotype	N	Abnormal BP events/participants	Abnormal BP prevalence, %	Adjusted OR (95% CI)	P value
Phenotype 1	342	75/342	21.93	Reference	—
Phenotype 2	781	381/781	48.78	1.88 (1.25-2.84)	<0.01
Phenotype 3	719	455/719	63.28	3.16 (2.03-4.92)	<0.01
Phenotype 4	294	242/294	82.31	4.83 (2.83-8.23)	<0.01

Phenotypes were derived without using blood pressure outcomes and were ordered from the most favorable to the most adverse mean standardized metabolic-adiposity profile. Abnormal blood pressure was defined as prehypertension or hypertension. Odds ratios compare each phenotype with phenotype 1 and are adjusted for age, sex, smoking status, alcohol drinking status, regular exercise, heart rate, HbA1c, LDL cholesterol, eGFR, uric acid, and log-transformed hsCRP.

### 3.6 Subgroup and sensitivity analyses

**Figure 6:**
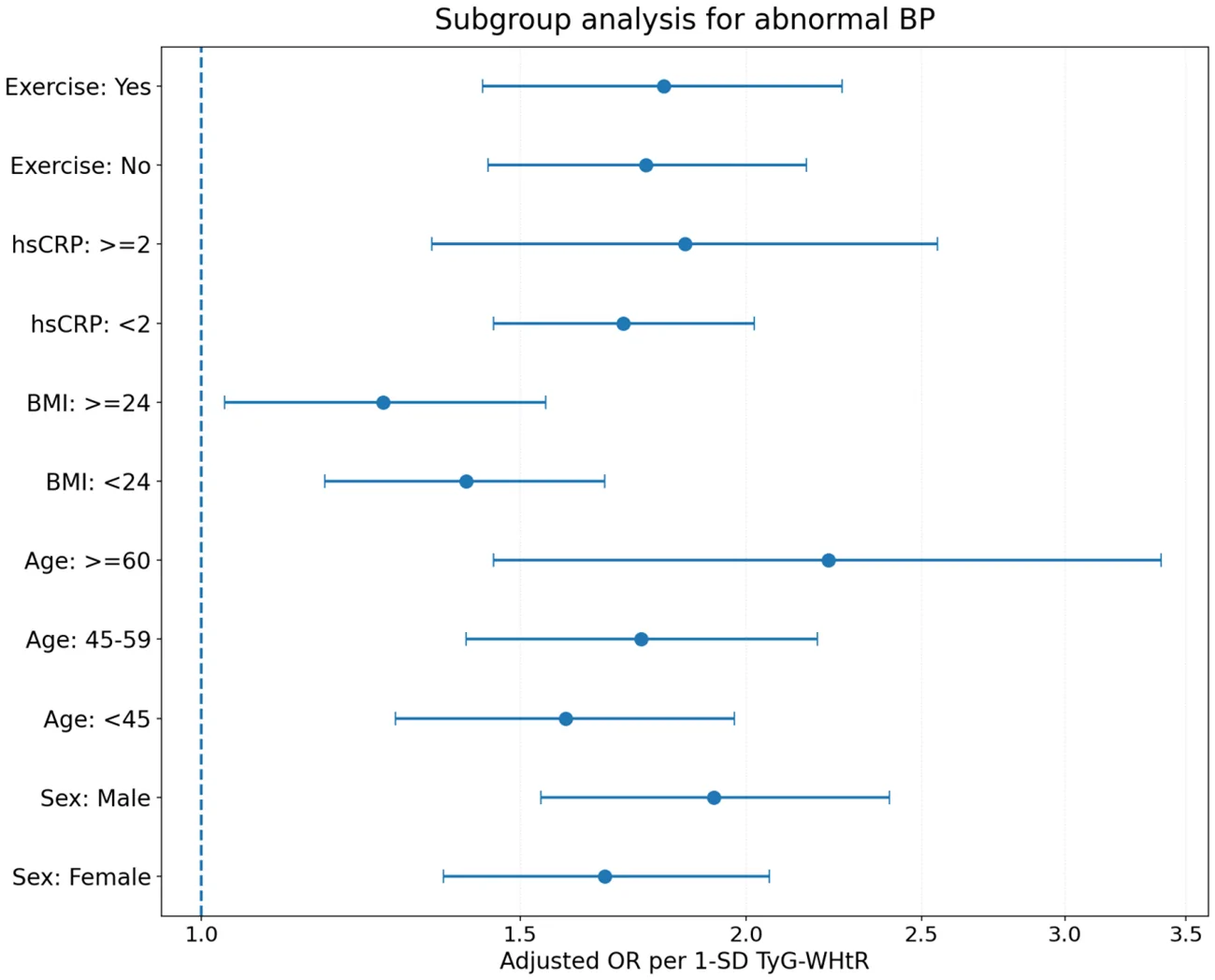
Subgroup analysis of TyG-WHtR and abnormal blood pressure.

Subgroup analyses using TyG-WHtR as a representative integrated metabolic-adiposity index showed directionally consistent positive associations across sex, age, BMI, hsCRP, and exercise strata (**Figure 6**). Sensitivity analyses excluding participants with hsCRP >10 mg/L and excluding the single implausible systolic-diastolic blood pressure record showed materially unchanged results. After additionally excluding 446 participants with self-reported known hypertension and/or antihypertensive medication use, 1, 690 participants and 707 abnormal blood pressure events remained. Associations persisted for AIP [OR, 1.37 (1.18-1.60)], CMI [OR, 1.29 (1.11-1.49)], CVAI [OR, 1.91 (1.58-2.31)], METS-IR [OR, 1.70 (1.45-2.00)], TyG-WHtR [OR, 1.59 (1.36-1.85)], BRI [OR, 1.46 (1.27-1.68)], and LAP [OR, 1.38 (1.19-1.59)]; all P values were <0.01. The WWI estimate was attenuated and no longer statistically significant (OR, 1.12 (0.99-1.27); P = 0.08) (**Table 6**). Continuous-outcome linear models also showed that higher AIP, CMI, CVAI, METS-IR, TyG-WHtR, BRI, WWI, and LAP were associated with higher systolic blood pressure, diastolic blood pressure, and mean arterial pressure.

**Table 6:** Sensitivity analyses for representative indices and abnormal blood pressure.

Sensitivity set	Index	N	Events	OR (95% CI)	P value
Primary data	Atherogenic index of plasma	2136	1153	1.49 (1.29-1.71)	<0.01
	Cardiometabolic index	2136	1153	1.46 (1.23-1.72)	<0.01
	Chinese visceral adiposity index	2136	1153	2.17 (1.80-2.62)	<0.01
	METS-IR	2136	1153	1.91 (1.63-2.24)	<0.01
	TyG-WHtR	2136	1153	1.76 (1.52-2.05)	<0.01
	Body roundness index	2136	1153	1.57 (1.37-1.80)	<0.01
	Weight-adjusted waist index	2136	1153	1.15 (1.02-1.29)	0.02
	Lipid accumulation product	2136	1153	1.59 (1.35-1.87)	<0.01
Excluding hsCRP >10 mg/L	Atherogenic index of plasma	2133	1150	1.48 (1.29-1.71)	<0.01
	Cardiometabolic index	2133	1150	1.45 (1.23-1.71)	<0.01
	Chinese visceral adiposity index	2133	1150	2.17 (1.80-2.61)	<0.01
	METS-IR	2133	1150	1.91 (1.62-2.24)	<0.01
	TyG-WHtR	2133	1150	1.76 (1.52-2.05)	<0.01
	Body roundness index	2133	1150	1.57 (1.37-1.80)	<0.01
	Weight-adjusted waist index	2133	1150	1.15 (1.02-1.29)	0.02
	Lipid accumulation product	2133	1150	1.59 (1.35-1.86)	<0.01
Excluding implausible SBP<DBP	Atherogenic index of plasma	2135	1152	1.48 (1.29-1.71)	<0.01
	Cardiometabolic index	2135	1152	1.46 (1.23-1.72)	<0.01
	Chinese visceral adiposity index	2135	1152	2.17 (1.80-2.62)	<0.01
	METS-IR	2135	1152	1.91 (1.62-2.24)	<0.01
	TyG-WHtR	2135	1152	1.77 (1.52-2.06)	<0.01
	Body roundness index	2135	1152	1.58 (1.38-1.81)	<0.01
	Weight-adjusted waist index	2135	1152	1.15 (1.02-1.30)	0.02
	Lipid accumulation product	2135	1152	1.59 (1.35-1.87)	<0.01
Excluding known hypertension/antihypertensive medication use	Atherogenic index of plasma	1690	707	1.37 (1.18-1.60)	<0.01
	Cardiometabolic index	1690	707	1.29 (1.11-1.49)	<0.01
	Chinese visceral adiposity index	1690	707	1.91 (1.58-2.31)	<0.01
	METS-IR	1690	707	1.70 (1.45-2.00)	<0.01
	TyG-WHtR	1690	707	1.59 (1.36-1.85)	<0.01
	Body roundness index	1690	707	1.46 (1.27-1.68)	<0.01
	Weight-adjusted waist index	1690	707	1.12 (0.99-1.27)	0.08
	Lipid accumulation product	1690	707	1.38 (1.19-1.59)	<0.01

Primary models were repeated after excluding hsCRP >10 mg/L, after excluding one implausible systolic-diastolic blood pressure record, and after excluding participants with self-reported known hypertension and/or antihypertensive medication use. Specific antihypertensive classes, doses, duration, adherence, and separate diuretic use were unavailable.

## 4 Discussion

In this hospital-based cross-sectional cohort of adults undergoing routine health examinations, prehypertension and hypertension were characterized by a broad adverse cardiometabolic profile. Participants with higher blood pressure categories were older and had greater general and central adiposity, higher glucose and HbA1c levels, higher triglyceride-rich and non-HDL lipid burden, lower HDL cholesterol, higher uric acid and hsCRP, and higher values across multiple derived indices. These findings reinforce the concept that blood pressure elevation in routine screening populations is embedded within a multidimensional metabolic-adiposity phenotype rather than occurring as an isolated hemodynamic abnormality.

The most prominent signals were observed for indices that integrate visceral or central adiposity with insulin-resistance or lipid information. CVAI, TyG-WC, TyG-BMI, METS-IR, and TyG-WHtR showed strong and consistent associations with abnormal blood pressure and hypertension. These indices may outperform single variables because they summarize complementary components: glycemia, triglyceride-rich lipoproteins, HDL cholesterol, BMI, waist circumference, and visceral adiposity distribution. This is biologically plausible because insulin resistance and visceral adiposity can promote sympathetic activation, renal sodium retention, endothelial dysfunction, arterial stiffness, inflammatory signaling, and renin-angiotensin-aldosterone system activation (9, 10).

Atherogenic lipid indices also remained associated with blood pressure status. AIP and TG/HDL-C may reflect triglyceride-rich lipoprotein metabolism and small dense LDL propensity, whereas Castelli risk indices capture cholesterol-fraction balance (23–25). Non-HDL cholesterol and remnant cholesterol represent residual atherogenic cholesterol burden (27–29). The observed association with blood pressure status is consistent with the clustering of dyslipidemia, insulin resistance, obesity, and hypertension within cardiometabolic risk syndromes.

The quartile and restricted cubic spline analyses add clinical interpretability. The highest quartiles of CVAI, METS-IR, and TyG-WHtR were associated with approximately four- to five-fold higher odds of abnormal blood pressure compared with the lowest quartiles, with clear trend P values. Spline analyses suggested statistically significant overall relationships for all representative indices, while strong nonlinearity was mainly evident for CMI. These results support the use of these indices as graded risk markers rather than binary cut points, although cutoffs may still be useful for pragmatic screening after external validation.

Mixture and phenotype analyses provided a deeper view of risk-factor clustering. Cardiometabolic, adiposity, atherogenic, and integrated novel-index mixtures were all positively associated with abnormal blood pressure and hypertension. The rerun unsupervised analysis separated participants into four progressively adverse metabolic-adiposity phenotypes, with abnormal blood pressure prevalence increasing from 21.93% in the most favorable phenotype to 82.31% in the most adverse phenotype. The corresponding adjusted odds increased stepwise across phenotypes. Because blood pressure outcomes were not used in model fitting or phenotype ordering, this gradient supports the clinical coherence of the metabolic-adiposity clustering while remaining an exploratory, internally derived finding that requires external replication.

The predictive modeling results should be interpreted carefully. Cross-validated AUROC improved from the basic model to conventional-biomarker, integrated-index, and full models, but the absolute improvement was modest. Independent test-set machine-learning models did not consistently outperform full or penalized logistic regression. This observation aligns with broader clinical prediction evidence showing that machine learning does not automatically improve performance over well-specified regression models (40, 41). Therefore, the machine-learning component is best viewed as an explanatory and robustness analysis rather than evidence for a deployable screening tool. The SHAP analysis strengthened the clinical coherence of the findings by identifying CVAI, age, fasting plasma glucose, waist circumference, eGFR, uric acid, hsCRP, LDL cholesterol, smoking status, and TyG-WC as influential features.

This study has several strengths. It used an independent hospital-based cohort with more than 2, 000 adults, no missing values in the analytic dataset, routine clinical variables that are readily obtainable in real-world health-examination centers, a broad panel of published composite indices, conventional regression, nonlinear analysis, mixture analysis, incremental discrimination, calibration, decision-curve analysis, supervised machine learning, SHAP interpretation, unsupervised phenotyping, subgroup analysis, and sensitivity analysis. The analysis also avoided using systolic and diastolic blood pressure as machine-learning predictors because those variables define the outcome.

Several limitations must be acknowledged. First, the cross-sectional design precludes causal inference and temporal interpretation. Second, blood pressure was derived from a single health-examination visit. The routine workflow generally used one reading, with repeat measurement only when the result was markedly discordant with self-reported history; it did not universally obtain duplicate readings and average them. The device manufacturer and model were not retained, a uniform rather than arm-circumference-specific cuff was documented, and no separate record of caffeine, smoking, or exercise restriction was available beyond the fasting protocol. Repeated office, home, or ambulatory blood pressure measurements were not available. Third, known hypertension and antihypertensive medication use were self-reported, and detailed drug classes, doses, duration, adherence, and separate diuretic use were unavailable. Although exclusion of participants with known hypertension and/or antihypertensive medication use yielded broadly consistent associations, residual medication-related confounding remains possible. Participants using glucose-lowering or lipid-lowering medications were excluded, which should also be considered when assessing generalizability. Fourth, the dataset did not include sodium intake quantification, sleep apnea assessment, psychosocial stress scales, insulin assays, renin-aldosterone biomarkers, or imaging-derived visceral fat. Chronic psychological stress may influence cardiometabolic risk through neuroendocrine, inflammatory, and behavioral pathways, but it was not measured in this cohort (44). Fifth, this was a single-center hospital-based cohort from Shandong, China, which may limit generalizability. Sixth, calibration was imperfect, no external validation was available, and the phenotype analysis was internally derived. Future multicenter longitudinal studies should incorporate standardized repeated blood pressure measurements, validated device information, detailed medication data, external validation, and incident hypertension outcomes.

## 5 Conclusion

In adults undergoing routine health examinations, atherogenic, cardiometabolic, and adiposity composite indices derived from routine anthropometric and biochemical variables were associated with prehypertension and hypertension. CVAI, TyG-WC, TyG-BMI, METS-IR, TyG-WHtR, AIP, CMI, BRI, LAP, non-HDL cholesterol, and remnant cholesterol provided interpretable information beyond basic demographic and lifestyle variables. These composite indices are not diagnostic substitutes for direct blood pressure measurement. Rather, they may help identify clustering of cardiometabolic risk that could prompt comprehensive lifestyle assessment and preventive counseling, while the present study did not evaluate the effectiveness of any intervention (45). Cross-sectional associations require cautious interpretation and prospective external validation.

## Data Availability

The original contributions presented in the study are included in the article/**Supplementary Material**. Further inquiries can be directed to the corresponding authors.
