# Supplementary material for: Atherogenic, cardiometabolic, and adiposity composite indices for identifying prehypertension and hypertension: conventional regression and explainable machine learning
Source: Front Endocrinol (Lausanne). 2026 Aug 25;17:1924726. doi: 10.3389/fendo.2026.1924726 (PMC13549880; doi:10.3389/fendo.2026.1924726)

Supplementary Figure S1. Participant flowchart.

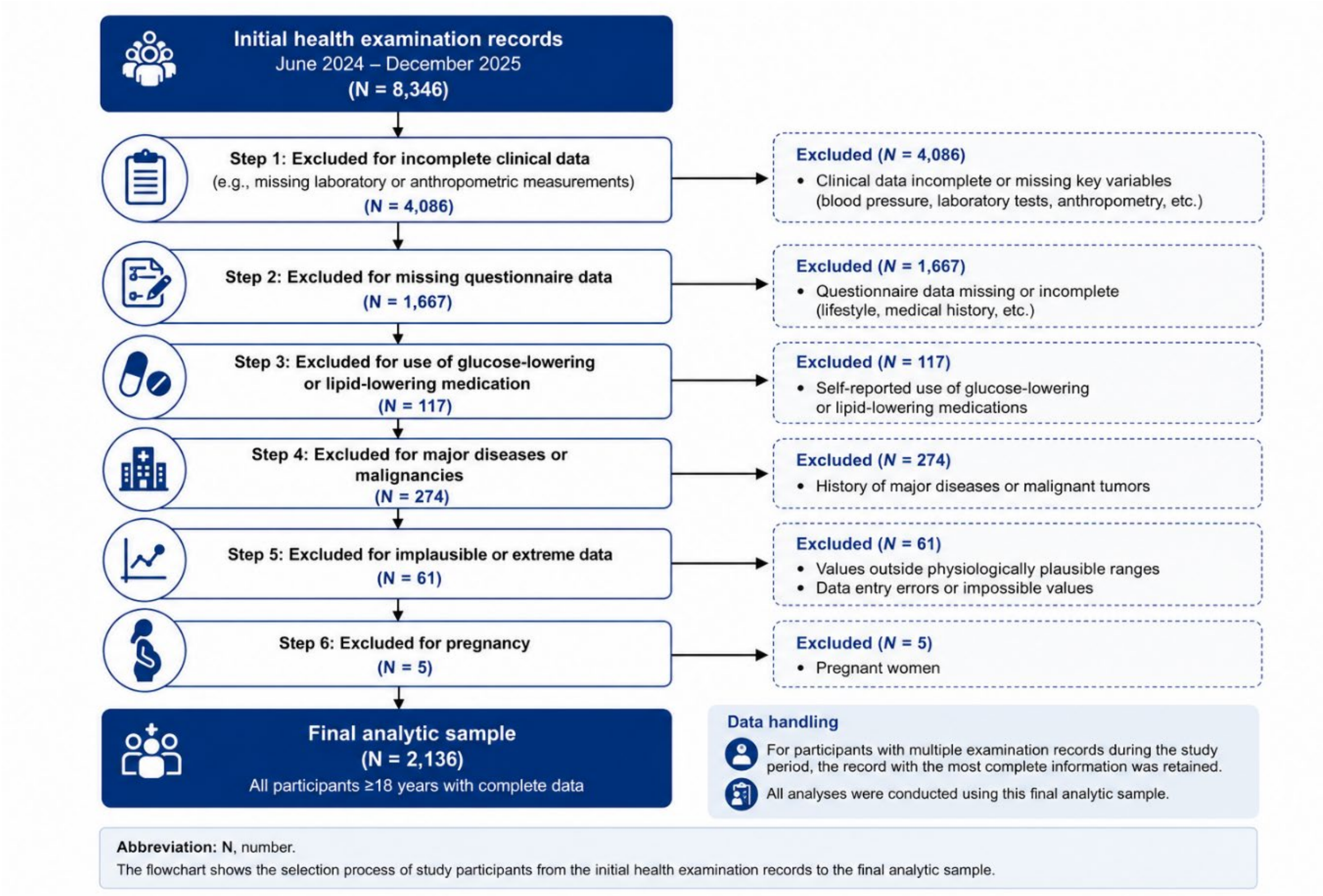

Supplement: Supplementary file 1 [file Image1.pdf]
